# Buyang Huanwu Decoction Ameliorates Poststroke Depression via Promoting Neurotrophic Pathway Mediated Neuroprotection and Neurogenesis

**DOI:** 10.1155/2017/4072658

**Published:** 2017-03-08

**Authors:** Lin Luo, Shuhua Deng, Jian Yi, Sainan Zhou, Yan She, Baiyan Liu

**Affiliations:** ^1^Hunan University of Chinese Medicine, Changsha, Hunan 410208, China; ^2^Yiyang Medical College, Yiyang, Hunan 413000, China

## Abstract

*Objective.* The aim of the present research is to investigate the therapeutic effect of Buyang Huanwu Decoction (BHD) in poststroke depression (PSD) animal model and illustrate its underlying mechanism via promoting neurotrophic pathway mediated neuroprotection and neurogenesis.* Methods.* To induce PSD rat model, isolation housed rats that received middle cerebral artery occlusion (MCAO) surgery successively suffered from chronic mild stress (CMS) treatment for consecutive twenty-one days. Meanwhile, rats were correspondingly given vehicle, BHD, and fluoxetine. Then, neurologic function was scored and depressive-like behaviors were assessed by sucrose preference test, locomotor activity, novelty-suppressed feeding test, and forced swim test. Thereafter, the neuroprotection and neurogenesis related molecular markers and signaling were detected.* Results.* We firstly observed a significant neurological function recovery and antidepressants effect of BHD after MCAO together with CMS treatment. Our study also found that treatment with BHD and fluoxetine can significantly rescue neurons from apoptosis and promote neurogenesis in the CA3 and DG regions in the hippocampus. Notably, BHD and fluoxetine treatment can activate BDNF/ERK/CREB signaling.* Conclusion.* The results suggest that BHD is a promising candidate for treating PSD. Its curative effects can be attributed to neurotrophic pathway mediated neuroprotection and neurogenesis.

## 1. Introduction

Poststroke depression (PSD) is among the most frequent neuropsychiatric consequences of stroke. The comorbid depression in stroke survivors is particularly prevalent, affecting approximately a third of individuals [1]. It negatively impacts stroke outcome with increased morbidity and mortality and poorer functional recovery [2]. Although PSD are attracting more and more attentions with a high incidence, there is still lack of effective therapeutic.

Mental distress associated with physical disability may contribute to the development of PSD [2]. Induction of depression by exposing isolation housed rats to chronic mild stress (CMS) after experimental stroke is the most common used model to mimic the clinical features of PSD [3–5]. This model not only induces depressive-like symptoms, which can be considered as the consequence jointly induced by MCAO and CMS [6], but also results in worse functional outcomes after stroke [4]. Currently, underlying mechanisms of PSD are associated in part with neuronal loss and impaired neurogenesis in the ischemic lesion and secondary degenerative changes [7]. Neurotrophin is not only essential for the development and neuronal plasticity of the CNS and for neuronal survival [8], but also widely implicated in psychiatric diseases [9]. Brain-derived neurotrophic factor (BDNF) has been demonstrated to play a role in both protection and recovery of functions after stroke [10, 11]. Moreover, abundant evidences provide the basis for the neurotrophic hypothesis of depression which associates decreased BDNF expression with depression and increased BDNF expression with antidepressant action, effects possibly mediated by alterations in neurogenesis [12–15]. In addition, accumulating evidences indicate that regulating the neurotrophic pathway may contribute to the functional recovery and antidepressant treatment in PSD models [3, 16, 17]. Therefore, it is plausible that the neurotrophic pathway may play a pivotal role in PSD treatment.

Buyang Huanwu Decoction (BHD) is a classic traditional Chinese medicine (TCM) herbal prescription which has been clinically used to treat stroke for centuries in East Asia. Abundant evidences have demonstrated its beneficial effects in ameliorating stroke-induced neurological dysfunctions in both experimental animal models and clinical therapeutics [18–23]. BHD rescues neurons from both oxygen glucose deprivation/reperfusion and MCAO insults [21]. Moreover, it has been demonstrated to promote neural differentiation and proliferation in ischemic penumbra [18, 24, 25]. Additionally, BHD has been observed to upregulate BDNF expression in infarct brain [26–28]. Therefore, we hypothesized that BHD might display an antidepressant effect in PSD animal model besides its neuroprotection. Thus, the aim of the present research is to investigate the therapeutic effects of BHD in PSD animal model and illustrate its underlying mechanism via promoting neurotrophic pathway mediated neuroprotection and neurogenesis.

## 2. Materials and Methods

### 2.1. Animals

Ninety male SpragueDawley rats, weighting 220–250 g at the beginning of the experiments, were purchased from SLAC Jinda, Hunan, China. Rats were group-housed with lights on from 7:00 to 19:00 until behavioral tests beginning and provided with ad libitum access to maintenance diet and water. The experiment was carried out according to the “Principles of Laboratory Animal Care” (NIH publication number 86-23, revised in 1996) and China legislation for the use and care of laboratory animals. All efforts were made to minimize animal suffering during experiments. The protocols were approved by the committee for the Care and Use of Laboratory Animals of Hunan University of Chinese Medicine, Changsha, China.

### 2.2. Focal Cerebral Ischemia

Rats were anesthetized with sodium pentobarbital (100 mg/kg, i.p.). A silicon-coated 4-0 monofilament was inserted in the left internal carotid artery and the monofilament was advanced to occlude the left middle cerebral artery (MCA). The monofilament was withdrawn 30 min after occlusion.

### 2.3. Chronic Mild Stress (CMS)

After MCAO surgery, all groups were kept in adjacent cages in the same area and rats in the MCAO group did not receive any stress and also had free access to food and water. Rats in the MCAO + CMS groups were isolation housed and underwent the chronic mild stress procedure from 2 days after MCAO.

CMS procedure was conducted according to Willner et al. with slight modifications [29]. A total of 7 different stressors were arranged in order for 21 consecutive days to induce a depressive-like state. The stressors included food and water deprivation (21 h), behavior restrictions (30 min), circadian rhythms reversal (24 h), 45° cage tilt (24 h), shaking on a shaking bed (200 Hz for 5 min), soiled cage (100 mL of water spilled onto the bedding, 23 h), and electric shock (0.9 mA, 15 s × 8 times). Each animal received one randomly scheduled stress per day and the same stressor did not appear consecutively. Each stressor appeared three times during the CMS procedure.

### 2.4. Drugs Preparation and Administration Procedure

BHD was prepared according to the prescription reported in Yi Lin Gai Cuo, which was published in 1820. The prescription is composed of Huangqi (*Astragalus membranaceus*, 120 g), Guiwei (*Radix Angelica sinensis*, 8 g), Chishao (Radix Paeoniae Rubra, 4.5 g), Honghua (*Carthamus tinctorius*, 3 g), Chuanxiong (*Ligusticum wallichii*, 3 g), Taoren (*Prunus persica*, 3 g), and Gandilong (dry* Pheretima aspergillum*, 3 g). To maintain the consistency of the herbal chemical ingredients, all components were obtained from the original sources and extracted according to standards listed in the National Pharmacopoeia of China. All materials were identified by the TCM professionals at Hunan Academy of Chinese Medicine (HACM). The voucher specimens were also deposited at HACM. The decoction was made by boiling the mixture in distilled water at 100°C for 30 min twice. The drug solution was then vacuum-cooled and dried to give the drug powder, which was dissolved in distilled water at a final concentration of 2.0 g/mL (equivalent to dry weight of raw materials).

Fluoxetine hydrochloride was obtained from Lilly (Suzhou, China).

Rats were assigned to five groups (*n* = 18 each) in a quasirandom manner. Initial random group assignments were adjusted using baseline sucrose consumption test to control for a preference bias. Rats in each group, respectively, received orally water (Sham, MCAO and PSD groups), fluoxetine hydrochloride (Flu group, 1.8 mg/kg), and Buyang Huanwu Decoction (BHD group, 12.8 g/kg, equivalent to the dry weight of the raw materials) from the beginning of CMS until the end of the behavioral test.

### 2.5. Assessment of Neurological Deficit Score

The neurological deficit score was assessed according to Kofler et al. [30] by three examiners blinded to the treatment groups on days 7, 14, and 21 during CUMS. The following neurological deficit scoring system was used: 0, no motor deficits (normal); 1, forelimb weakness and torso turning to the ipsilateral side when held by tail (mild); 2, circling to the contralateral side but normal posture at rest (moderate); 3, unable to bear weight on the affected side at rest (severe); and 4, no spontaneous locomotor activity or barrel rolling (critical). Animals with no significantly neurological deficit observed 60 min after occlusion period were removed from further study.

### 2.6. Behavioral Task

#### 2.6.1. Sucrose Preference Test (SPT)

A sucrose preference baseline test was performed one day before MCAO surgery. The sucrose preference test was conducted 21 days after CMS. After a 23 h food and water deprivation, rats were presented with 1% sucrose solution and water for 1 h. Liquid intakes were measured by weighing the bottles at the end of each test. All tests were conducted in the home cage of rat to minimize exogenous interferences. The sucrose preference (SP) was calculated according to the following ratio: SP = sucrose intake/(sucrose intake + water intake) [31].

#### 2.6.2. Locomotor Activity (LA)

Locomotor activity was tested using a black box (100 × 100 × 40 cm) housed in sound-attenuated rooms. Rats were initially placed in the center of the test box and their behaviors were video-recorded for 3 minutes. The travelled distance of each rat was analyzed by a SMART 2.5 soft (Panlab S.L.U., Barcelona, Spain).

#### 2.6.3. Novelty-Suppressed Feeding (NSF) Test

Hyponeophagia refers to the inhibition of feeding produced by exposure to novelty. The NSF test was performed according to An et al. [32]. After 48 h food deprivation, rats were put into a new plastic rat cage from the corner. Thirty equal sized food pellets were placed in the center of the box and the first latency of food intake in a 5-min period was manually recorded.

#### 2.6.4. Forced Swim Test (FST)

One day before the test day, rats were preexposed to water in a glass cylinder (15 cm in height and 10 cm in diameter) for 5 min. On the test day, behavior was recorded using a digital camera for 5 min and the immobile time was manually scored by two blinded observers.

### 2.7. Immunohistochemistry and Hematoxylin and Eosin Staining

Histopathology was performed after completion of behavioral tests. Rats were sacrificed after deep anesthetization with sodium pentobarbital (100 mg/kg, i.p.) and transcardial perfusion with cold heparinized saline, followed by perfusion of 4% paraformaldehyde. Brains were removed and postfixed overnight in paraformaldehyde, before being dehydrated and embedded in paraffin. Brains were cut into 5 *μ*m thick sections in the coronal plane and stained with hematoxylin and eosin (HE). For immunohistochemistry, brain sections were rehydrated and blocked with 2% normal goat serum for 2 h, then incubated with rabbit polyclonal anti-Nestin antibody (1 : 100, Boster, China), and subsequently reacted with the biotinylated secondary antibody. Histostain-SP kits (Zymed) were used to visualize the immune complexes according to the manufacturer's instructions. Images were captured on a Leica optical microscope. The staining density was measured in integrated optical density (IOD) in three random images from each brain section.

### 2.8. TUNEL Staining

DNA fragmentation was performed by In Situ Cell Death Detection Kit (Keygen Biotech, China). Coronal brain sections (5 mm thick) were stained according to the methods provided by the manufacturer. Apoptotic cells were stained brown due to the binding of dUTP enzyme to their fragmented DNA. At last, all sections were mounted with a DAB horseradish peroxidase color development kit. Apoptotic neurons and total neurons in the ischemic cortex were counted for five fields per section under high-power magnification (400x) by a blinded manner. The apoptotic ratio was calculated as follows: apoptotic ratio = apoptotic neurons/total neurons × 100%.

### 2.9. Bromodeoxyuridine (BrdU) Labeling

To label the proliferating cells, six rats in each group received a successive 5 days' injection of BrdU (100 mg/kg, i.p., Sigma-Aldrich) after MCAO. Then, free-floating brain sections were denatured in 1 M HCl for 30 min at +60°C. Preincubation was carried out for 1 h at room temperature with 5% goat serum in 0.1 M PBS containing 1% Triton-X-100. Subsequently, sections were incubated with the rat anti-BrdU monoclonal primary antibody (1 : 100, Chemico, USA) overnight at +4°C. Finally, HRP conjugated secondary antibody was incubated for 30 min at room temperature and followed by DAB horseradish peroxidase color development.

### 2.10. RT-Quantitative PCR

For quantitative real-time PCR analysis, human premessenger RNA sequence was obtained from the NCBI (National Center for Biotechnology Information) AceView program (https://www.ncbi.nlm.nih.gov/AceView/). All primers were designed with Primer 5.0 software and the primers used in our research were as follows: NESTIN, sense 5′-AGG ATG TGG AGG TAG TGA GA-3′, antisense 5′-TGG AGA TCT CAG TGG CTC TT-3′; BDNF, sense 5′-GTG ACA GTA TTA GCG AGT GGG-3′, antisense 5′-TAT CCT TAT GAA CCG CCA GCC-3′; CREB, sense 5′-TCA GCC GGG TAC TAC CAT TC-3′, antisense 5′-TCT CTT GCT GCT TCC CTG TT-3′; *β*-actin, sense 5′-AGT GCG ACC TGG ACA TCC G-3′, antisense 5′-TGG CTC TAA CAG TCC GCC TAG-3′. Total RNA was isolated using RNA-Solv Reagent (OMEGA). Reverse transcription was performed with 2 *μ*g RNA using ReverTra Ace (TOYOBO) and Oligo(dT)18(TaKaRa). qRT-PCR was carried on SYBR® Premix Ex Taq (TaKaRa) using ViiA™ 7 Real-Time PCR System (Thermo). Reaction procedures were as follows: an initial step at 94°C for 4 min, 40 cycles of 94°C for 40 s, and 60°C for 30 s.

### 2.11. Western Blotting

The hippocampus was promptly dissected out on ice and homogenized in ice-cold RIPA buffer containing a protease inhibitor cocktail. The protein concentration was determined using a BCA protein assay kit (Thermo scientific, USA). Denatured proteins were separated by SDS-PAGE gel (CWBIO, China), thereafter transferred onto polyvinylidene-difluoride (PVDF) membrane. Membranes were incubated overnight at 4°C with either rabbit anti-ERK1/2 (1 : 500, Boster, China), rabbit anti-p-ERK1/2 (1 : 500, Boster, China), or rabbit anti-p-CREB (1 : 500, Boster, China). Subsequently, membranes were incubated with HRP conjugated anti-rabbit second antibodies (1 : 1000, Beyotime, China) for 2 h at room temperature. The immunoreactive bands were visualized by using ECL chemiluminescence detection kit (CWBIO, China) and the intensity of the blots was analyzed using Image Pro plus 6.0.

### 2.12. Statistical Analysis

All analyses were performed using SPSS version 16.0 (Chicago, IL, USA). Values were expressed as means ± standard error of the mean (SEM), and statistical significance was set at *P* < 0.05 in all of the evaluations. The data were analyzed with one-way or repeated measures analysis of variance (RM ANOVA) where statistically appropriate. Post hoc multiple group comparisons were made using a Tukey post hoc test. The results of the analysis of these data were only reported when a significant difference was observed.

## 3. Results

### 3.1. BHD Ameliorated PSD-Induced Neurological Deficits and Depressive Symptoms

**Figure 1:**
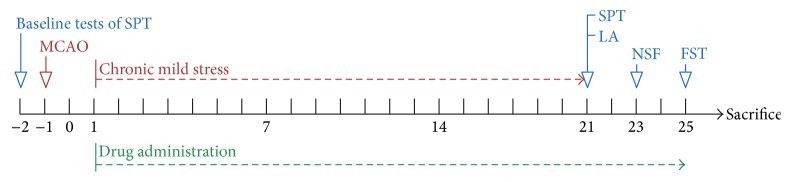
Time schedule of experimental procedures.

**Figure 2:**
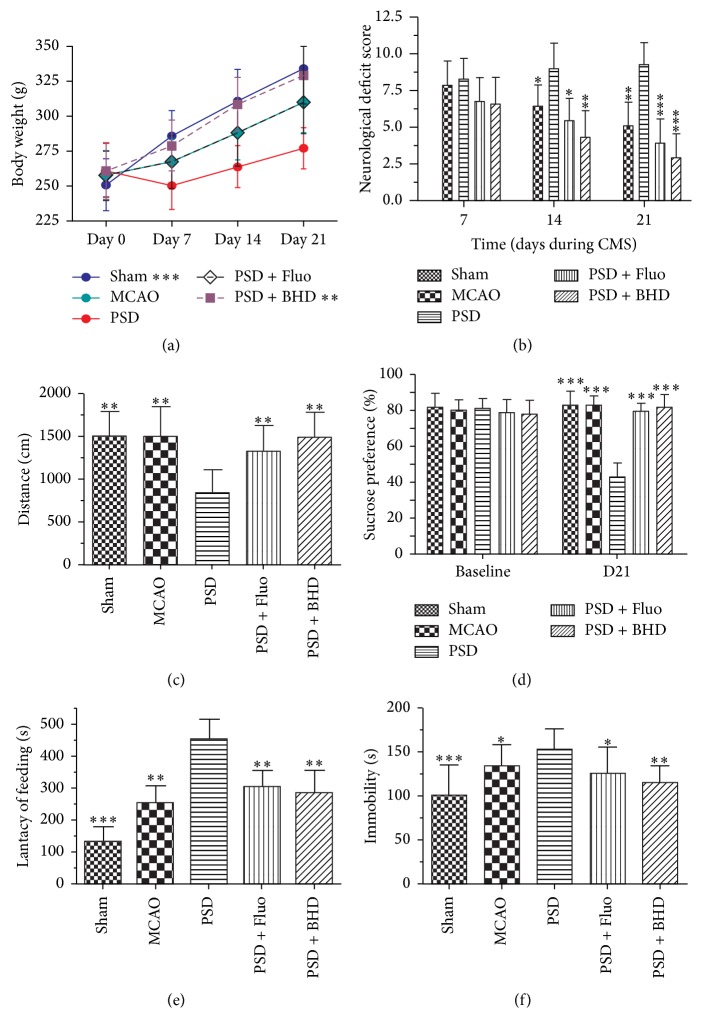
BHD ameliorated PSD-induced neurological deficits and depressive symptoms. Subpart (a) showing the weight alterations during 21 days of CMS after MCAO surgery; subpart (b) is the quantification of neurologic scores; subpart (c): results of locomotor activity; subpart (d): results of sucrose preference test; subpart (e) is the results of novelty-suppressed feeding test; subpart (f) is the results of forced swimming test. Data are presented as mean ± SD (*n* = 18 each group), ^*∗*^*P* < 0.05, ^*∗∗*^*P* < 0.01, ^*∗∗∗*^*P* < 0.001 indicate significant difference compared with the PSD group.

Figure 1 showed the time schedule of experimental procedures. To determine the effects of BHD on neural function recovery, the body weight of rats was firstly observed weekly during the CMS phase. As shown in Figure 2(a), MCAO + CMS induced a severe decrease in body weight from the first week. The administration of BHD and fluoxetine significantly attenuated the body weight reduction. Then, we evaluated the neurological deficit scores of rats at the end of CMS phase. Consistently, PSD rats exerted a delayed functional recovery in a time depended manner. Obviously, rats that have undergone MCAO + CMS treatment displayed severely neurological deficit compared with other groups, even compared with MCAO single treated group twenty-one days after CMS exposure (see Figure 2(b)). However, BHD and fluoxetine treatment significant ameliorated the neurological deficit. Thereafter, we conducted several behavioral tests to determine the antidepressant effects of BHD on PSD.  Figure 2(c) represents the total distance the rats performed in locomotor activity test. PSD rats displayed a significantly decreased locomotor activity, whereas BHD and fluoxetine treatment reversed the reduction of locomotor activity. As shown in Figure 2(d), PSD group displayed a decreased sucrose preference ratio than other groups. Consistently, PSD rats showed remarkably increased latency of feeding in novelty-suppressed feeding test and immobility time during forced swimming test. We observed that rats treated with BHD and fluoxetine showed better behavior performance compared with PSD rats in these depressive-like behavior tests. The results suggested that BHD treatment can significantly ameliorate PSD-induced neurological deficits and depressive symptoms.

### 3.2. BHD Rescued Neurons from PSD-Induced Neuronal Apoptosis in the Hippocampus and Cortex

**Figure 3:**
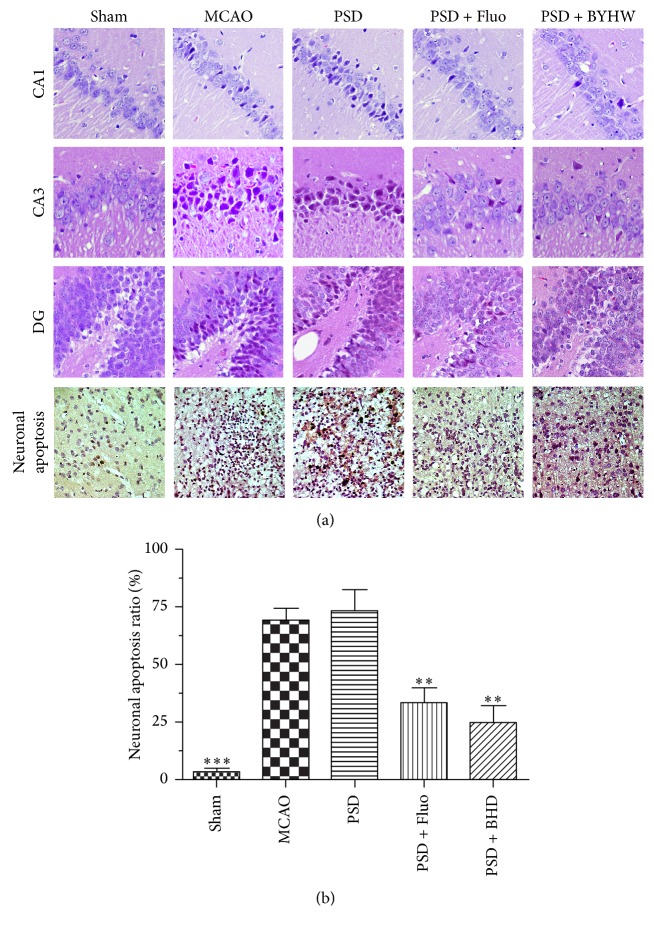
BHD rescued neurons from PSD-induced neuronal apoptosis in the hippocampus and cortex. Subpart (a) showing the effect of BHD treatment on morphological changes in different brain regions after PSD by hematoxylin-eosin and TUNEL staining; subpart (b) is the statistic of TUNEL-positive cells. Data are presented as mean ± SD (*n* = 6 each group), ^*∗∗*^*P* < 0.01, ^*∗∗∗*^*P* < 0.001 indicate significant difference compared with the PSD group (scale bar = 50 *μ*m).

Then, we evaluated the neuroprotective effect of BHD against PSD-induced neuronal apoptosis. We, respectively, conducted H&E and TUNEL staining in the hippocampus and cortex. The HE staining could visually show the histological changes in neurons in different brain regions (Figure 3(a)). In the sham operated rat, neurons in the CA1, CA3, and DG regions of the hippocampus were round or oval in shape and the nuclei were clear. After MCAO, a large number of apoptotic neurons with karyopyknosis, cell gaps, and debris could be observed in vehicle group. BHD and Fluo treatment alleviated the symptoms of apoptosis in different degrees. Moreover, cell apoptosis is one of the major causes for ischemia-induced delayed neuronal death. The severity of neuronal apoptosis within the ischemic penumbra was evaluated by apoptotic index, defined as the percentage of TUNEL-positive cells in neurons. Significantly more TUNEL-positive cells were found in MCAO and PSD groups, while BHD and Fluo treated groups displayed less TUNEL-positive cells.

### 3.3. BHD Promoted Neurogenesis in the Hippocampus

**Figure 4:**
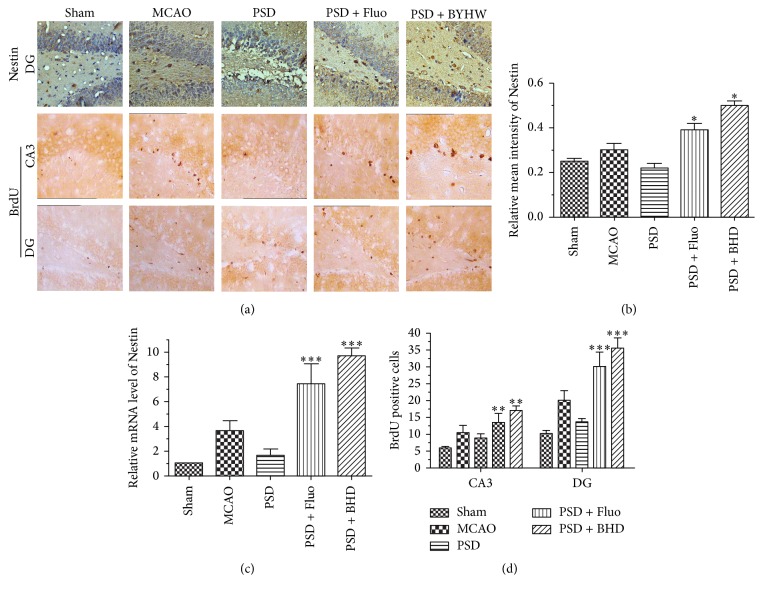
BHD promoted neurogenesis in the hippocampus. Subpart (a) showing the alterations of Nestin and BrdU expression in the hippocampus by immunohistochemical staining; subpart (b) is the result of relative intensity analysis of Nestin; subpart (c) is the statistic of BrdU positive cells; subpart (d) is the result of Nestin mRNA expression. Data are presented as mean ± SD (*n* = 6 each group), ^*∗*^*P* < 0.05, ^*∗∗*^*P* < 0.01, ^*∗∗∗*^*P* < 0.001 indicate significant difference compared with the PSD group (scale bar = 50 *μ*m).

**Figure 5:**
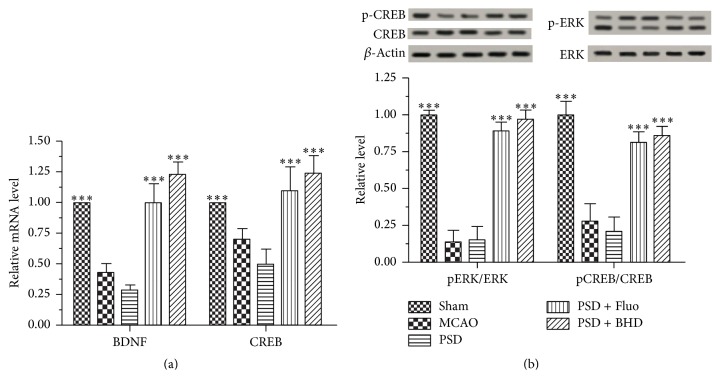
Effects of BHD treatment on the expression levels of ERK/CREB signaling in the hippocampus. Subpart (a) is the result of BDNF and CREB mRNA expression; subpart (b) is the expression levels of pERK, ERK, pCREB, and CREB proteins. Data are presented as mean ± SD (*n* = 3 each group), ^*∗∗∗*^*P* < 0.001 indicate significant difference compared with the PSD group.

To determine whether BHD exerts its antidepressant effects via promoting neurogenesis in the hippocampus, we detected the alterations of neurotrophin signaling. Hippocampal neurogenesis has been demonstrated to be required for antidepressant-like behavior [14].  Figure 4(a) showed the immunohistochemical staining of Nestin in the DG region and BrdU positive neurons in the CA3 and DG regions. As shown in Figure 4(b), the relative intensity of Nestin was significantly higher in BHD and Fluo groups compared with PSD group. Accordingly, the relative mRNA expression level of Nestin was remarkably elevated after BHD and Fluo treatment compared with PSD group (see Figure 4(d)). As shown in Figure 4(c), the number of BrdU positive neurons was significantly higher in the CA3 and DG regions in BHD and Fluo treated groups compared with PSD group. Moreover, we observed an increased mRNA expression level of CREB and BDNF in BHD and Fluo treated groups (see Figure 5(a)). Consistently, as shown in Figure 5(b) both the MCAO and PSD group displayed marked declines in p-ERK/ERK and p-CREB/CREB ratio. However, the expression levels of p-ERK and p-CREB were remarkably elevated after BHD and Fluo treatment. The results indicated that BHD possibly exerts its antidepressant effects via activating neurotrophic signaling cascade mediated neurogenesis in the hippocampus.

## 4. Discussion

Exposure of animals to unpredictable chronic mild stress (CMS) after ischemic stroke is a primary way to elicit experimental poststroke depression. Animals subjected to a series of unpredictable mild stressors will suffer from major depressive disorders involving anhedonia, anxiety, and behavioral despair [29]. It has been further demonstrated that CMS after stroke exacerbates the neurological deficits and depressive-like symptoms [4–6]. Moreover, exposure to chronic stress after stroke can enhance neuronal loss or degeneration and impair neurogenesis [33, 34]. Consistent with previous findings, we found that rats exposed to 21 days of CMS after MCAO surgery remarkably developed core features of depression. In addition, MCAO + CMS rats exhibited more severely neurological deficits after CMS exposure. Several articles have reported that BHD could ameliorate neurological dysfunction after ischemic stroke [18, 21, 22]. Its neuroprotection should be attributed to reducing infarct volume, rescuing neurons from neuronal apoptosis, promoting angiogenesis, and antioxidative properties [18, 20–22]. Our data indicated that BHD as well as fluoxetine treatment not only significantly improved function recovery and decreased neuron apoptosis, but also notably attenuated depressive-like symptoms.

It has been demonstrated that the hippocampus is implicated in the pathogenesis of depression [35]. Although current evidence indicates that adult hippocampal neurogenesis may not play a crucial role in the development of depression, it may contribute to ameliorate depression-like behaviors [33]. Psychological stress and depressive-like behaviors are associated with the impairment of plasticity in the adult brain such as proliferation of neural progenitor cells [36]. Besides, neurogenesis in dentate gyrus of the hippocampus plays a pivotal role in stroke recovery, and this neurogenesis also promotes recovery from depression [2]. We observed that MCAO surgery led to upregulation of adult neurogenesis. However, MCAO + CMS rats showed decreased neuronal proliferation and differentiation compared with MCAO rats. Therefore, it is evident that the severe depressive-like behaviors exerted in PSD rats are closely associated with inhibited neuronal proliferation and differentiation in the hippocampus. Several articles indicate that antidepressants may exert their behavioral effects by increasing neurogenesis in the dentate gyrus [33, 37, 38]. Fluoxetine therapy has been reported to have a beneficial effect on poststroke emotional disorders and cognitive function [39, 40]. In accordance with previous findings, we noticed that both BHD and fluoxetine treatment obviously increased the expression level of Nestin and the number of BrdU positive cells. Thus, the results demonstrated that the amelioration effects of BHD and fluoxetine on PSD should be attributed to its neuroprotection and promotion of neurogenesis in the hippocampus [14].

Moreover, a leading hypothesis of depression suggests that neurotrophic factors are critical in mediating the behavioral responses to antidepressants [15, 34, 41]. Brain-derived neurotrophic factor (BDNF) is a neurotrophin that has been linked to the viability of neurons, cognition, and mood-related behaviors [42–44]. Additionally, several studies reported that serum concentrations of BDNF decrease in PSD patients and BDNF may play an important role in the pathogenesis of PSD [16, 17]. The ERK is the member of the mitogen activated protein kinase (MAPK) family and necessary for cell growth, differentiation, and survival [45]. BDNF is an upstream regulator of the ERK cascade and the phosphorylated ERK has been proposed as an intracellular signaling mechanism mediating antidepressant efficacy [46]. Ultimately, ERK signal cascades activate CREB gene expression which is thought to be responsible for neurogenesis and neural development [47, 48]. Notably, our observation indicated that BHD and Fluo were likely to rescue neurons from apoptosis and promote neurogenesis via modulating BDNF/ERK/CREB signaling.

In conclusion, we observed a significant neurological function recovery and antidepressants effect of BHD after MCAO together with CMS treatment. The results of the present study suggest that BHD is a promising candidate for treating PSD. Its curative effects can be attributed to neurotrophic pathway mediated neuroprotection and neurogenesis.

## Abbreviations

BDNF: Brain-derived neurotrophic factor
BHD: Buyang Huanwu Decoction
BrdU: Bromodeoxyuridine
CMS: Chronic mild stress
MCAO: Middle cerebral artery occlusion
PSD: Poststroke depression.
